# Medical Opinion and Movement

**Published:** 1908-10-24

**Authors:** 


### Medical Opinion and Movement.

THE maldescended testis, the correct treatment of which still excites controversy and is by no means settled, is the subject of two papers in the *Annals of Surgery*. Dr. W. B. Coley summarises his personal series of 128 cases, giving after histories in all those he has been able to trace. He is very adverse to sacrifice of the gland, and, as a rule, to operation of any sort under the age of 8 to 12 years. He agrees with Eccles that the evidence is flimsy on which is based the old notion that a maldescended is more liable than a normal testis to be affected by sarcoma. The excretory value of the gland to the patient may be little or nothing; but, nothwithstanding this, it is held that it may be of great use in developing the male characteristics: in other words, a testis may form no spermatozoa and yet fulfil its functions of internal secretion. As to methods of operation he recommends free opening up of the inguinal canal, as by Bassini's method; thorough freeing of the testis from adhesions or peritoneal bands, with sacrifice of veins if necessary; bringing the testis into the scrotum, and suture of the canal without transplantation of the cord. It is claimed that there has been but one recurrence of the accompanying hernia, and that in only two cases of the series was it found necessary to remove the gland; both of the patients were adults. Dr. Coley is strongly of opinion that it is unjustifiable to operate on children at two years old, as some surgeons have recommended, as in a large proportion of these the testis will ultimately reach the scrotum unaided. He is also averse from the use of any suture, splint, or other mechanical contrivance for fixing the organ in the scrotum. Dr. Coley concludes with the somewhat ambiguous advice that no case of double undescended testis should be allowed to reach the age of puberty.

MR. STARR'S contribution is chiefly devoted to the method of operation, and to a description of a plan of silver-wire splintage which he has found to answer well as a retentive measure. Like Dr. Coley, he condemns the removal treatment, but he is willing to allow that in cases where the maldescended testis is near the internal abdominal ring it may be advisable to restore it to the abdomen, a plan which has been advocated in this country as a routine by Mr. E. M. Corner. In ordinary cases he dissects the cord free from its coverings, and, if necessary to obtain increased length, sacrifices the spermatic and cremasteric arteries, but not the artery to the vas. He then passes chromic catgut sutures through the tunica albuginea and fastens them to a piece of plaited silver wire some two or three inches long. The wire ends are then made to protrude through the lower end of the scrotum and bent at right angles; to make assurance doubly sure, a horsehair stitch is passed through the openings from which the wire emerges, is made to catch up the tunica, and is then brought out again and tied over the projecting wire ends. The upper end of the wire is sutured with chromic catgut to the periosteum on the os pubis. On the twelfth day the silver splint is removed by pulling on the projecting ends and easily comes away. The description of this operation sounds rather terrifying, but Mr. Starr says the results are most satisfactory. He does not touch on the question of the most suitable age for the performance of the operation.

AT the recent International Surgical Congress several interesting subjects were discussed. We have already alluded to the lengthy debate on the surgical treatment of hepatic and biliary disorders. No less interesting was the discussion on anæsthetics. As was to be expected, spinal and local anæsthesia held the van, the only paper on general anæsthesia being read by Vallas, who did not support his main contention, that ether is the anæsthetic *par excellence*, by any new arguments or facts. Several communications were read on the subject of lumbar anæsthesia, and these proved definitely how much ground this method has won during the last few years. On the whole the consensus of opinion was in favour of the method, and individual speakers drew from a large experience of cases. Thus Jonnesco of Bucharest uses spinal anæsthesia not only for operations on parts below the umbilicus but for operations on the thorax, head, and neck, and injects strychnine as well as stovaine. The majority of speakers agreed that stovaine was not the ideal anæsthetic, and gave the preference to tropacocaine, a verdict in which everyone who has had practical experience of the two drugs must concur. The dangers of lumbar anæsthesia were frankly pointed out by those who are not enamoured of it, and as frankly admitted by the supporters of the method. Thus it may produce general mischief, and may set up grave kidney trouble. Most of these objections apply with special force to stovaine, but even with tropacocaine great care has to be taken. The risk of sepsis and the introduction of epithelial cells into the canal is always present, and "rachistovainisation" is scarcely likely to supplant local and ether anæsthesia in general practice.

KEHR, of Halberstadt, who claims to have had a personal experience of 4,000 cholelithiatic patients, introduced a discussion on the subject of the surgical treatment of gall-stones and liver diseases. He divides the indications for operation into absolute and relative. Absolute indications are acute and chronic empyema of the gall-bladder, and "obstruction of the bile duct not yielding rapidly to medical treatment." The relative indications vary in each case and must be decided by the surgeon on each separate occasion. On the whole Kehr appears to hold operation justifiable in most instances where the patient belongs to the poorer classes, and cannot be expected to submit himself to the prolonged course of treatment at spas or otherwise which can be prescribed for his more wealthy fellow-sufferer. In agreement with most modern authorities, Kehr strongly insists that the operation of choice is a cholecystectomy. It is the only really radical operation, and it is an operation which is not more fatal and scarcely more difficult than an ordinary cholecystostomy. A great point in its favour is that the after treatment is shortened and that recurrences are unknown.

---

FOLLOWING Kehr came Alessandri, who drew attention to the surgical aspects of liver abscess, and pointed out that, in Italy at least, the disease is increasing in frequency. Payr, of Greifswald, discussed tumours of the liver and gall-bladder, and stated that he had found the Wassermann serum reaction of definite value in the diagnosis of early gumma of the liver. Ribera, of Sans, discussed the treatment of hydatids of the liver based on a series of 79 cases under his own care, and laid stress on the difficulty in differential diagnosis of hydatid tumours. Eosinophilia he had found by no means constant, and he has given up attempting to diagnose a case of hydatid disease as such, preferring to make a diagnosis by exclusion. Very little that is new was brought out in the discussion on biliary carcinoma. All the speakers agreed as to its malignancy and the difficulty of early diagnosis, and all were agreed upon the advisability of radical and thorough operation.

---

A MUCH more interesting discussion followed the notes on the surgical treatment of hepatic cirrhosis. The ordinary Talma operation and the modification of it proposed by Narath, of Heidelberg, have now firmly established themselves as legitimate surgical procedures, but as yet there does not seem to be any definite agreement as to their indications. The results of the Talma operation are usually extremely uncertain, and in consequence a large number of ingenious and complicated operations have been devised. The severe operation which bears Eck's name, and which has been often performed on animals, has now been done with success on one patient by Vidal, but it is hardly a modification that will meet with general approval. Pengniez, of Amiens again advises splenectomy in cases of intractable cirrhosis, holding that the disease is caused by the elaboration of toxins by the spleen. In two cases he has been favourably impressed with the results of this operation, his patients having progressed very satisfactorily since their spleens have been excised.

---

THE International Congress of Thalassotherapeutics has just taken place at the beautiful seaside resort of Abbazia. Over 250 physicians attended the meeting and many interesting addresses were delivered by which it was endeavoured to systematise the principles and methods of treatment of various ailments by the resources peculiar to the seaside—sea bathing, sea baths, sea air and not the least of them abundance of sunshine, or, more technically, *insolation*. It is rather surprising to find that several papers were devoted to the beneficent effect of the sea air and of sea bathing in asthmatic conditions, for it is the experience of many physicians that the sea air is not well borne by asthmatic subjects, although it may be beneficial in individual cases. Dr. Diem dealt with the subject of floating sanatoria. Such institutions are already in existence in France and Germany. Dr. Diem's idea is to have a number of steamers fitted with the necessary arrangements for treating medical and surgical cases on the open sea. The vessel should not follow any special route but should be able to stay anywhere most suitable according to meteorological conditions. From a study of weather charts, the distribution of the winds and the hours of sunshine, Dr. Diem is led to the opinion that the Adriatic and middle of the Mediterranean would offer the best positions for such vessels. With the exception of a few days during November the floating sanatoria could remain in the open sea in these parts without fear of storms.

---

DR. J. BATNAND calls attention in the *Revue des Maladies de la Nutrition* to a form of uterine prolapse, which he calls "neurasthenic." Few local lesions are noticeable in this form of prolapsus; the perineum remains intact, but there is a simple relaxation of the utero-sacral ligaments. The condition may be termed neurasthenic, from the fact that many neurasthenic troubles are usually associated with it, such as gastric dilatation, enteroptosis, arterial hypo-tension, muscular asthenia, and physical and moral lassitude. The only treatment of use in cases when the patient has an enormous prolapse, and is long past the menopause, consists in the application of a cup pessary to the prolapsing parts, the instrument being kept in place by a simple T bandage. In less marked cases, where the perineum, although insufficient, is firm, all that is necessary for the patient to do is to take an injection of tannin every morning, and then insert tampons of sterilised wool into the vagina. These must be retained until bedtime. If the uterus be normal, or only slightly increased in size, and provided that the menopause has not set in, there is hope of a permanent cure being effected by such treatment unless the trouble is of long-standing. Uterine displacements must be first corrected, by pessaries if necessary. If there is a cystocele as well as the uterine prolapse, a tampon of wool must be used in addition to the pessary. In cases of recent onset, after a few months the utero-sacral ligaments are sufficiently reposed to regain some of their former tone. General treatment by means of tonics, strychnine, and baths, will be of help in effecting increased tonus of the ligaments. Constipation must be carefully guarded against, as the effects of straining at stool are extremely bad. The patients must also be warned against allowing their bladders to become distended. A suitable belt will be of value in allowing the patient to walk and stand erect without danger. Such a one is that known in France as Glénard's.

---

AMONG the many factors probably entering into the ætiology of rickets the age of the parents has received little attention. The view has been held by some that children born of parents at either extreme limit of age are especially liable to the disease. Dr. P. Gelati has undertaken statistical inquiry at the children's clinic at Parma during the past four years with the object of ascertaining some data on this point. During this period 3,121 children have been examined, and of these 615 either were, had been, or became rickety, a proportion of 19.71 per cent. Of these 3,121 patients, part came from the country districts around and part were from the town, and there was a higher percentage of rickety children among the former than among the latter. The figures are: 1,779 town children with 307 cases of rickets, or a percentage of 17.26; and 1,215 country children with 301 cases of rickets, or a percentage of 24.77. The place of abode was not noted in the remaining 127 patients. The figures in regard to the age of the parents show a steady increase in the incidence of disease in proportion to the parents' age. For parents under 20 the incidence of the disease was as low as 11.6 per cent., and rose steadily to 19.2 per cent. for children of parents between 20 and 30, and further to 22.2 per cent. for the next decade, and 22.7 per cent. for children of parents between 40 and 50. For the same decade the proportion of rickets was always higher among the country than among the town children. The children were also divided into three classes according to whether they were exclusively breast-fed for six months or more, or partially so fed, or brought up altogether on artificial feeding. The proportion of cases of rickets increases from the first to the third class, showing the importance of the food factor; but given the same feeding the percentage of rickets is greater in proportion to the age of the parents.

---

DECHLORIDATION, or the elimination of sodium chloride from the diet, is a form of treatment long advocated in certain affections, especially of the kidneys, and reference has already been made to this method in these columns. Dr. Langstein has applied it to the treatment of obstinate cases of eczema in infants. He feeds them with a liquid food containing the same proportion of albumen, sugar, and fat as in milk, but with only a fifth part of the salts. Treated in this way the eczema disappears in the course of four to six weeks. The diet requires careful supervision, as in some cases it is not borne well and the child may lose in weight, but after a short period more salt may be added to the food without any return of eczematous signs. A further important observation of the same author is the great increase in the number of leucocytes in all infants affected with this disease. In some cases the proportion of eosinophiles amounted to 27 and 33 per cent., whereas normally it is not more than 1 per cent. in infants. It is impossible to decide whether this leucocytosis is due to some abnormal constitutional factor or simply dependent upon the local affection of the skin.

---

DR. W. B. HILL analyses in the *Quarterly Journal of Medicine* eight cases of idiocy of the type described over 40 years ago by Dr. Langdon Down as Mongolian. The ætiology of this peculiar condition, which is believed to be slightly more common in Britain than on the Continent, is not in any way connected with syphilis, alcoholism, or tuberculosis in the parents; but maternal ill-health during gestation seems to be frequently met with, and nearly all the mothers are over 40 and have had many (healthy) children. Mongolians form from 3 to 5 per cent. of all classes of idiots in this country, and are, Dr. Hill says, more numerous than cretins. Of the physical characteristics brachycephaly is one of the most constant, indeed, the breadth of the skull is almost equal to the length; the plane of the occiput tends to be parallel with that of the face and forehead. The fontanelles and sutures are slow to close. Langdon Down described straight and sparse brownish-black hair as one of the characteristic features of the patients, but Dr. Hill's cases present no peculiarity in this respect, nor is there any anomaly of the ears. The shape of the face is round; the cheeks are chubby and of a ruddy colour, the nose short with broad depressed bridge, and the palpebral fissures narrow, sloping downwards and inwards. Strabismus and nystagmus are not uncommon, epicanthus is frequent. The lower lip is often everted, and the tongue is nearly always protruded and fissured. The bones show retarded ossification, but no other lesions.

---

MENTALLY, Mongolian idiots display all grades of deficiency, but most of them are simply imbecile. They are apathetic and easy to manage, being good tempered and easily amused. Some observers have described special aptitude for drill, dancing, and music. They are never erotic or vicious, contrasting markedly thus with most other feeble-minded children. They acquire the power of speech very late, and also that of walking and of movement generally. Muscular power is feeble, but sight and hearing are good. The knee-jerk is often absent, and the plantar reflex extensor. One of the most peculiar features of the disease is the absolute uniformity of the type; owing to this a case can be recognised at once, and it is said that parents have great difficulty in picking out their own children on visiting days in institutions containing several of these patients. The pathology of the disease remains mysterious; no lesions have been constantly found otherwhere than in the brain, though congenital morbus cordis and other defects are not rare. The ductless glands present no demonstrable abnormalities. In the brain are found thinning of the cortex and rarefaction of the nerve cells, especially the large pyramidal cells. The prognosis of all cases is very bad, mentally and physically. Lung diseases, chiefly pneumonia, bronchitis, and tuberculosis, carry off most of the children before they reach puberty. No drug treatment is known which has any effect at all. Thymus and thyroid medication have been tried, and, as might be expected from the post-mortem findings, have been proved to be without value. The prospect of improving the patient's mental condition by pedagogic methods is also not a particularly brilliant one.

